# Family-based NGS panel testing of cardiopathies and arrhythmic syndromes

**DOI:** 10.3389/fgene.2025.1677311

**Published:** 2025-11-03

**Authors:** Hana Hrazderova, Jana Petrkova, Anna Crhova, Klara Herkommerova, Kvetoslava Mahutova, Julie Nejezchlebova, Lenka Petrkova, Lubos Boucek, Arpad Boday, Spiros Tavandzis

**Affiliations:** 1 Department of Medical Genetics, AGEL Laboratories, Novy Jicin, Czechia; 2 Agel Hospital Trinec-Podlesi, Trinec, Czechia; 3 Department of Internal Medicine I - Cardiology, University Hospital Olomouc, Olomouc, Czechia; 4 Department of Pathological Physiology, Faculty of Medicine and Dentistry, Palacky University Olomouc, Olomouc, Czechia; 5 Department of Pharmacology, Faculty of Medicine, Masaryk University, Brno, Czechia

**Keywords:** segregation analysis, cardiomyopathies, arrhythmic syndromes, risk alleles, next-generation sequencing

## Abstract

Hereditary forms of cardiovascular disease represent a highly heterogeneous group of disorders with a prevailing autosomal dominant inheritance pattern, incomplete penetrance, and variable expressivity. Segregation analysis can help elucidate the genetic aetiology of these diseases, which may be ambiguous within individual families, thereby allowing for a more accurate risk assessment of family members. In this study, we present an alternative approach to co-segregation studies based on comprehensive clinical and molecular genetic diagnostics as part of routine testing. Next-generation sequencing was performed in 58 individuals from 12 families, including asymptomatic individuals. Pathogenic sequence variants and variants of uncertain significance of genes related to cardiopathies and arrhythmic syndromes were identified in 7 families, and their segregation within these families was observed. All willing family members were tested extensively from the start of the diagnostic process, as opposed to testing only genes found in the proband. This method enabled faster risk stratification and clinical follow-up of at-risk family members, facilitating improved disease prevention and personalised patient management.

## 1 Introduction

Cardiovascular diseases represent a diverse group of pathologies, some of which affect the myocardium or the heart´s electrical conduction system. Genetic variability plays a role in many of these conditions (Kathiresan and Srivastava, 2012), which may exist as isolated clinical entities or be part of genetically determined syndromes (Prawitt and Eggermann, 2024). These disorders are primarily determined by genes with an autosomal dominant inheritance pattern, exhibiting incomplete penetrance and variable expressivity (Kathiresan and Srivastava, 2012).

Furthermore, the observed phenotype may also be influenced by other factors like phenocopies, modifier genes, and epigenetic factors such as DNA methylation and miRNAs. Local epigenetic modifications that modulate the response of the myocardium and tissue-specific regulatory mechanisms may also help explain the variability in phenotypes. Environmental factors such as physical exercise or psychological stress can further activate or suppress clinical manifestations in predisposed individuals (García-Padilla et al., 2024; Yan et al., 2024). This complex interplay of influences complicates diagnosis and underlines the importance of a personalised and multidisciplinary approach in the management of patients with inherited cardiac diseases.

Mendelian monogenic and oligogenic disorders are caused by rare genetic variants that tend to cluster in the affected families (Wilde et al., 2022). Segregation analysis is often considered a fast and accessible method for assessing the pathogenicity of variants of uncertain significance in routine diagnostics (Angelova et al., 2024). However, in cardiogenetics, its informativeness is limited due to incomplete penetrance, variable expressivity, and other aforementioned factors (Lacaze et al., 2021; Lukas Laws et al., 2022). To avoid misinterpretations, these aspects must be considered. Methods such as the Bayesian approach and LOD scores are commonly used to evaluate uncertain variants.

So far, segregation analysis applies only to specific families and is a time-consuming process due to standard procedures (Ylipää and Andersson, 2024). In cardiogenetics, the proband’s DNA is first analysed using Next-generation sequencing (NGS), followed by targeted Sanger sequencing of family members and retrospective cardiac evaluations based on the results (Stava et al., 2024).

This study aimed to optimize molecular genetic testing in families with cardiopathies and arrhythmic syndromes. The approach included comprehensive cardiac evaluations, genetic counselling, and NGS-based laboratory analysis in as many family members as possible, including asymptomatic individuals. The strategy of extensive testing from the start of the diagnostic process enabled faster and more accurate variant interpretation, improving clinical predictions and treatment decisions. Additionally, pathogenic variants linked to arrhythmic syndromes were identified in some patients originally tested for other cardiac conditions.

## 2 Materials and methods

### 2.1 Case presentation

This study included 58 individuals from 12 families with a cardiac burden. All probands and relatives underwent cardiological evaluation, and a provisional diagnosis was made (Supplementary Table S1), identifying cardiac rhythm disorders in four families and cardiomyopathies in six. DNA from peripheral blood samples was analysed using NGS panel testing, Sanger sequencing, or other molecular methods. Informed consent was obtained, and the study was approved by the Ethics Committee, following Good Clinical Practice and legal regulations.

### 2.2 DNA isolation, NGS

Peripheral blood samples were collected, and DNA was isolated, quantified, and quality-checked using a NanoDrop spectrophotometer and Qubit fluorometer (Thermo Fisher Scientific, Waltham, MA, United States ). NGS analysis was performed on NextSeq 550 (Illumina, San Diego, CA, United States) and DNBSEQ-G50 (Shenzhen, China) using a custom Twist panel targeting 717 clinically relevant genes linked to cardiomyopathies, arrhythmic syndromes, congenital heart disease, sudden cardiac death, RASopathies, cardiac defects, connective tissue disorders, and rare genetic diseases (Supplementary Table S2). The panel covered coding regions, intron overlaps (20–50 bp), untranslated regions (∼200 bp), and known regulatory variants. For further analysis, only variants with greater than 10× coverage and greater than 15% frequency in the sample were selected.

### 2.3 Bioinformatics analysis

Sequencing data from NextSeq 550 and MGIseq were aligned to the hg38 reference genome using the Burrows-Wheeler Alignment Tool (BWA MEM). Variant calling for single nucleotide polymorphisms (SNPs), insertions, and deletions was performed with Samtools, VarScan, and Pindel, generating Variant Call Format (VCF) files. Variant annotation was conducted using National Center for Biotechnology Information (NCBI) databases and the Ensembl Variant Effect Predictor (VEP), while CNV (Copy Number Variation) analysis was performed with the Genome Analysis Toolkit (GATK).

Identified variants were classified into five categories: pathogenic, likely pathogenic, variants of uncertain significance (VUS), likely benign, and benign, following ACMG guidelines (Walsh et al., 2024).

### 2.4 Statistical analysis

The analysis of data and the subsequent calculation of descriptive statistics were conducted utilising the R software (version 4.2.2, R Core Team, 2022). The ggplot2 package was employed for the generation of heat maps, while the ComplexHeatmap package was utilised for the visualisation of intersections of gene variants in patients. The descriptive statistics employed included medians and ranges, in addition to frequencies with percentages (n [%]).

## 3 Results

Complete panel assays were performed in 58 individuals from 12 families. Sequence variants in the following clinically relevant genes were identified in 7 families (41 patients): alpha cardiac actin 1 (*ACTC1*), ankyrin-2 (*ANK2*), calcium voltage-gated channel subunit alpha1C (*CACNA1C*), desmoglein-2 (*DSG2*), four and a half LIM domains 1 (*FHL1*), junction plakoglobin (*JUP*), potassium voltage-gated channel subfamily Q member 1 (*KCNQ1*), plakophilin-2 (*PKP2*), phospholamban (*PLN*), RNA binding motif protein 20 (*RBM20*), and sodium voltage-gated channel alpha subunit 5 (*SCN5A*). It was found that in some of the families, the identified variants segregated with the disease. The segregation analysis confirmed the already established pathogenicity of some variants, while also highlighting the possible clinical impact of rare variants of unclear significance.

**FIGURE 1:**
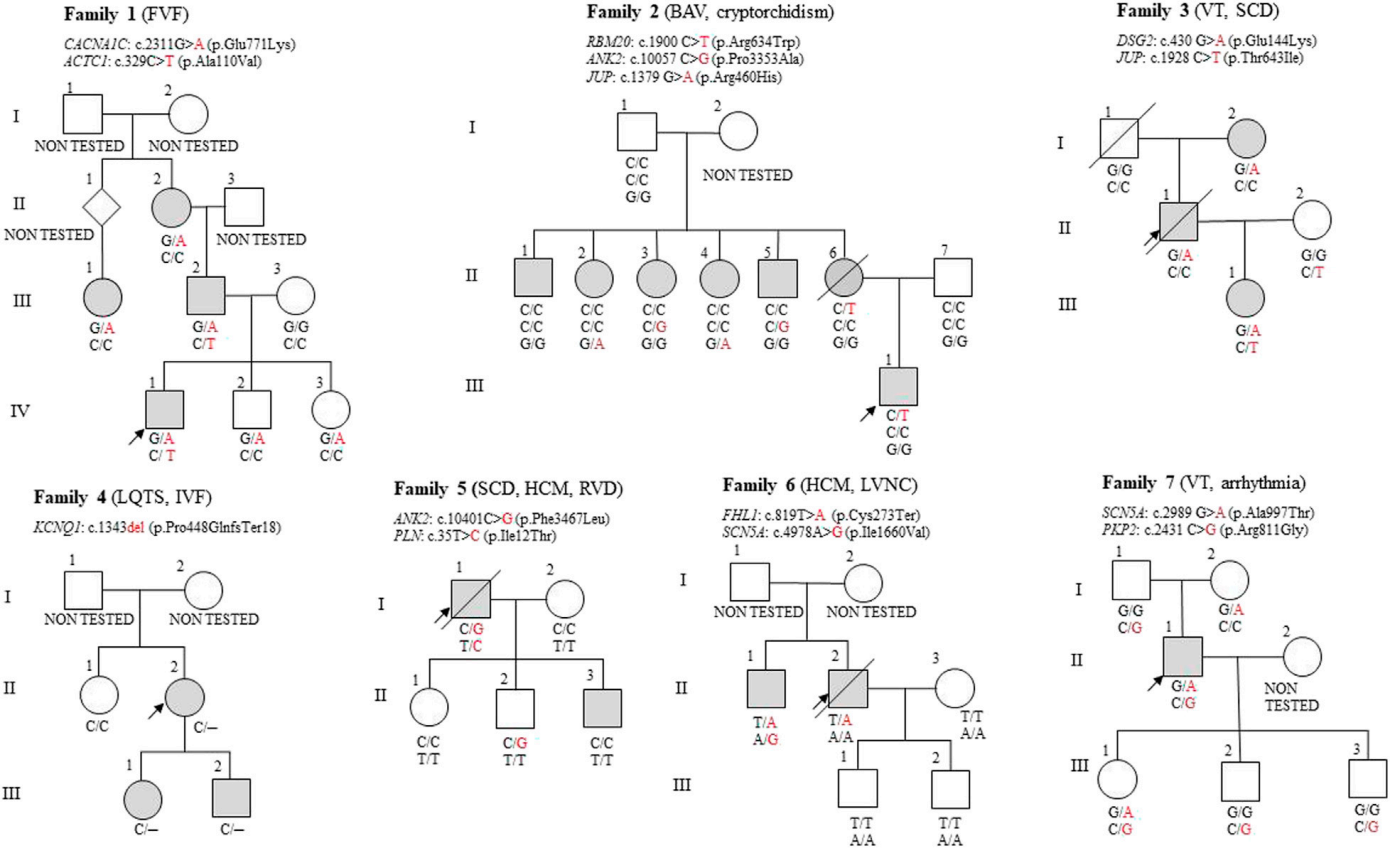
Pedigrees of the seven families described in this article. The proband is marked with a black arrow in each pedigree. A crossed-out symbol indicates that the person is deceased. The coloured symbol indicates the phenotypic expression of different types of heart disease. Diseases diagnosed in the proband are indicated in brackets. FVF, Familial Ventricular Fibrillation; BAV, Bicuspid Aortic Valve; VT, Ventricular Tachycardia; SCD, Sudden cardiac death; LQTS, Long QT syndrome; IVF, Isolated ventricular fibrillation; HCM, Hypertrophic cardiomyopathy; RVD, Right Ventricular Dilation; LVNC, Left Ventricular Non-Compaction.

**FIGURE 2:**
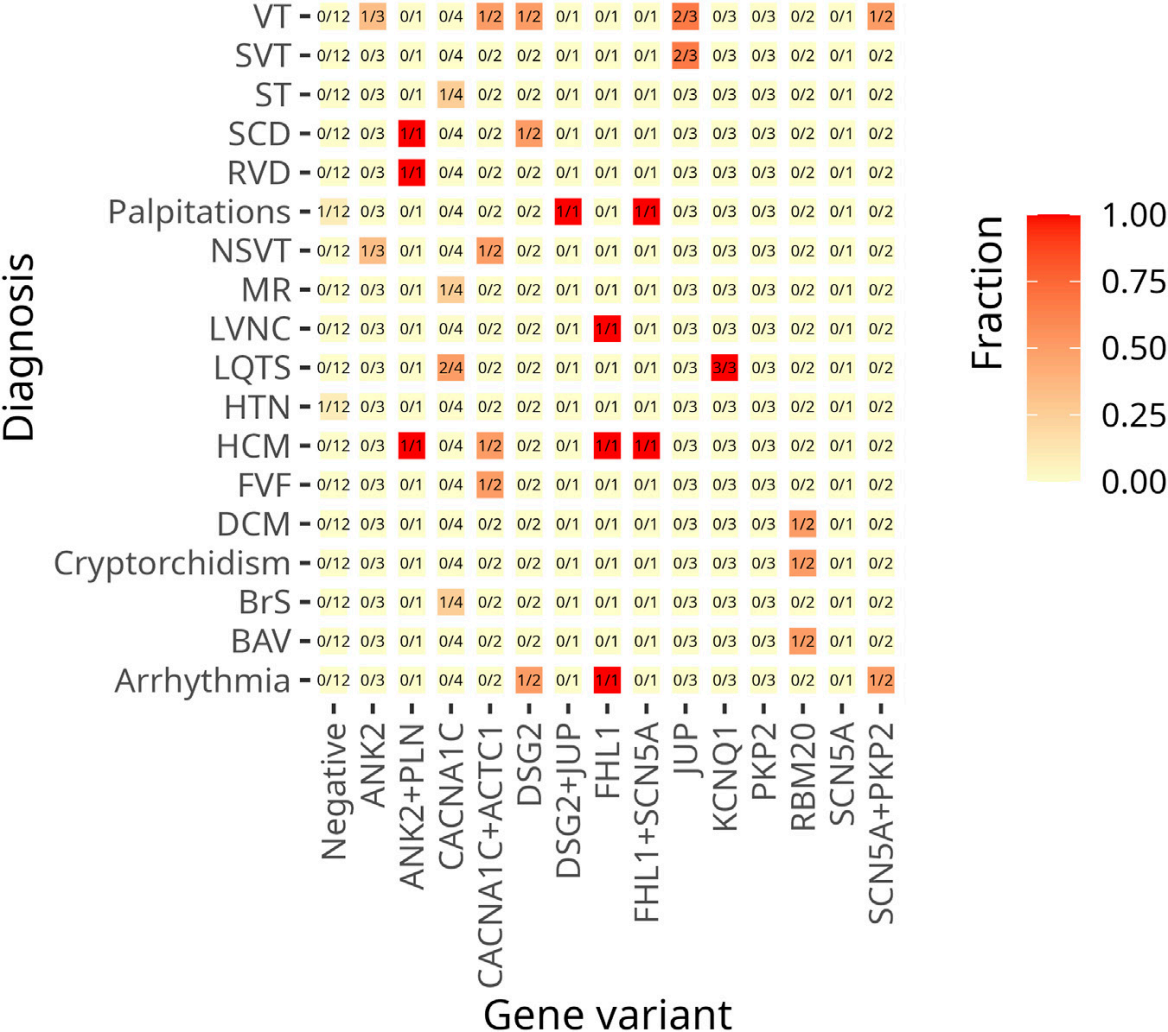
This heat map visualizes the frequency of cardiac diagnoses across genetic variants. The Y-axis lists diagnoses, while the X-axis represents genes with identified variants. The colour gradient reflects the proportion of patients with each diagnosis within a given genetic subgroup. The lighter shades (yellow) indicate lower frequencies, the darker shades (red) indicate higher frequencies. A value of 1.0 (dark red) indicates that all individuals with the variant have the diagnosis, whereas 0.0 (light yellow) indicates that no cases within the subgroup are affected. The numerical values in the cells indicate the proportion of affected individuals in each group of variants.

**FIGURE 3:**
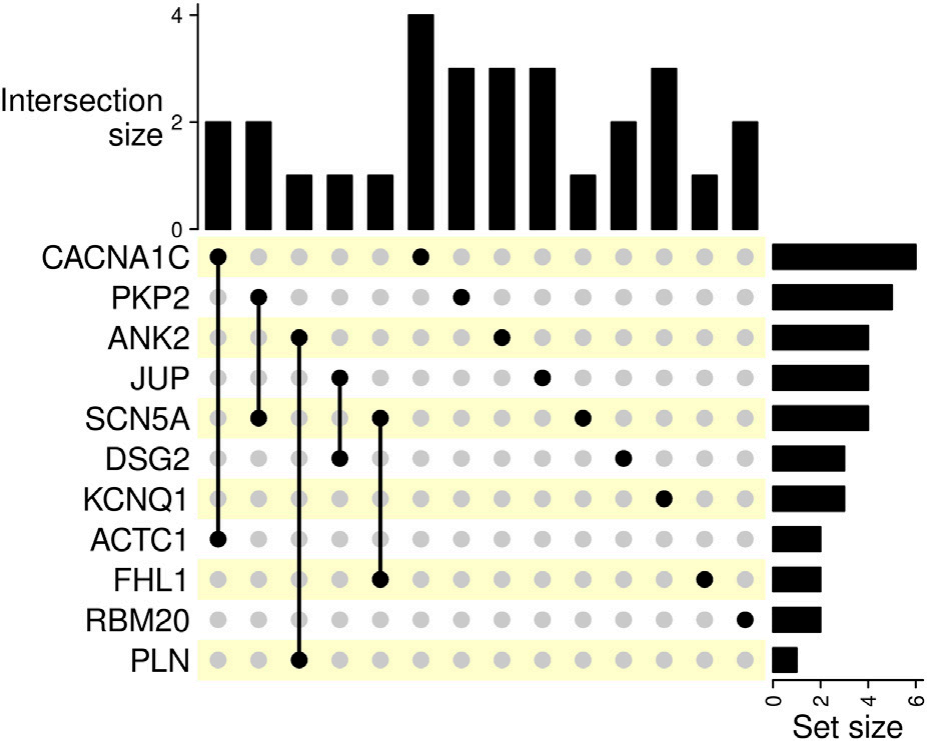
This UpSet plot visualizes intersections of gene variants, the intersection sizes, and set sizes. Bar charts on the right represent the number of occurrences of the corresponding gene variant in the dataset. The size of the intersections is indicated by bar charts on top.

For comprehensive information, including clinical data, genetic test results, patients’ characteristics, and variant distribution across the examined families, refer to Supplementary Table S1 in the Supplementary Material and Table 1. Detailed information on the distribution of variants within families is provided in the pedigrees (Figure 1). Figure 2 illustrates the frequency of diagnoses across genetic variants. Notably, diagnoses of sudden cardiac death (SCD), hypertrophic cardiomyopathy (HCM), and dilated cardiomyopathy (DCM) are strongly associated with variants in genes *DSG2*, *SCN5A*, *PKP2*, and *RBM20*. Figure 3 effectively illustrates the distribution of gene variants and co-occurrence patterns in the dataset.

**TABLE 1:** Characteristics of patients by gene variant.

Gene variant	Count	Age	Males (n [%])
Negative	12	47.5 (9–97)	7 [58.3 %]
*ANK2*	3	42 (13–52)	2 [66.7 %]
*ANK2*+*PLN*	1	39	1 [100 %]
*CACNA1C*	4	32 (25–81)	1 [25 %]
*CACNA1C*+*ACTC1*	2	44.5 (34–55)	2 [100 %]
*DSG2*	2	65 (49–81)	1 [50 %]
*DSG2*+*JUP*	1	15	0 [0 %]
*FHL1*	1	50	1 [100 %]
*FHL1*+*SCN5A*	1	45	1 [100 %]
*JUP*	3	50 (47–52)	0 [0 %]
*KCNQ1*	3	28 (26–53)	1 [33.3 %]
*PKP2*	3	18 (10–83)	3 [100 %]
*RBM20*	2	29 (13–45)	1 [50 %]
*SCN5A*	1	81	0 [0 %]
*SCN5A*+*PKP2*	2	35.5 (23–48)	1 [50 %]

Age is presented as median and range.

### 3.1 Family 1

The proband (IV-1) was diagnosed with ventricular fibrillation, required cardiopulmonary resuscitation (CPR), and received an implantable cardioverter-defibrillator (ICD). Genetic analysis identified an *ACTC1* variant c.329C>T (p.Ala110Val) and a *CACNA1C* variant c.2311G>A (p.Glu771Lys), both of uncertain significance.

The proband inherited both the *CACNA1C* and *ACTC1* variants from his father (III-2), who has HCM and non-sustained ventricular tachycardia. Furthermore, the proband´s paternal grandfather has a suspect anamnesis, considering he suffered and died from heart disease; however, he couldn´t be included in the analysis.

His paternal cousin, diagnosed with Long QT syndrome, carries the *CACNA1C* variant. This variant was also found in the proband´s asymptomatic siblings without Long QT syndrome diagnosis (IV-2, IV-3), with his sister being a professional athlete. The proband´s paternal grandmother (II-2) with sinus tachycardia and mitral regurgitation also carries the *CACNA1C* variant, though she does not have Long QT syndrome either.

An ACTC1 variant was identified in the proband and his father, both of whom presented with ventricular tachycardia. ACTC1, an autosomal dominant variant, is associated with hypertrophic and other cardiomyopathies (Greve et al., 2024). A segregating *CACNA1C* variant was identified across three generations in individuals with cardiac issues, suggesting a probable pathogenic effect. *CACNA1C*, inherited in an autosomal dominant manner, is linked to arrhythmic syndromes with incomplete penetrance (Cipriano et al., 2024), explaining why some carriers (e.g., the proband’s siblings) remain asymptomatic. A synergistic effect between *CACNA1C* and *ACTC1* may contribute to the severe phenotype observed in the proband and his father.

The proband’s brother (IV-2) and sister (IV-3) carry the *CACNA1C* variant, increasing their risk of heart disease due to family history. Although asymptomatic, regular cardiological monitoring is crucial, especially under high physical or psychological stress, to detect potential disease manifestations early.

### 3.2 Family 2

The proband (III-1), age 12, has a bicuspid aortic valve and cryptorchidism. A heterozygous *RBM20* variant c.1900C>T (p.Arg634Trp) was identified in the analysed family. This variant is strongly associated with DCM and has been confirmed as pathogenic in multiple segregation studies (Tukker et al., 2023; Das and Seth, 2021). However, the proband doesn´t show DCM symptoms so far.

The *RBM20* variant was inherited from the mother (II-6), who died of sudden cardiac death at 37 years. She was also diagnosed with left ventricular hypertrophy and dilatation during post-mortem examination. None of her five siblings (II-1 to II-5) carried the *RBM20* variant. These relatives had supraventricular heart rhythm abnormalities without cardiomyopathy associated with rare *JUP* and *ANK2* variants of uncertain significance.

The results lend support to the pathogenicity of the variant in the *RBM20* gene. Although the penetrance of this variant is incomplete in the 12-year-old proband so far, he has a higher risk of developing a full phenotype later in adulthood.

### 3.3 Family 3

The proband (II-1) died of sudden cardiac death due to ventricular tachycardia. Genetic analysis identified a *DSG2* sequence variant c.430G>A (p.Glu144Lys) of uncertain significance.

The proband inherited the variant from his mother (I-2), who was diagnosed with arrhythmias. His daughter (III-1), suffering from heart palpitations, carries both the *DSG2* variant inherited from her father and the *JUP* variant c.1928C>T (p.Thr643Ile) from her healthy mother (II-2).

Based on the available data, the c.430G>A p. (Glu144Lys) variant in the *DSG2* gene is likely to be pathogenic, especially in view of its segregation with disease over three generations. Family members should undergo regular cardiac monitoring and genetic counselling to minimise the risk of serious arrhythmias and other complications.

### 3.4 Family 4

The proband (II-2) with Long QT syndrome (LQTS), isolated ventricular fibrillation, and an ICD implant carries the pathogenic *KCNQ1* variant c.1343del (p.Pro448GlnfsTer18). This deletion was also found in his daughter (III-1) with LQTS and his son (III-2) with a prolonged QT interval, confirming its significant role in the family phenotype.

*KCNQ1* encodes a potassium ion channel and is a key gene in LQTS development (Giammarino et al., 2024). The c.1343del variant causes a frameshift and premature STOP codon formation, impairing channel function and prolonging the QT interval (Morgat et al., 2024).

Families carrying the c.1343del variant face a higher risk of LQTS, arrhythmias, and sudden cardiac death. Regular cardiac monitoring, genetic testing for relatives, and preventive treatment should be considered to minimize major cardiac risks.

### 3.5 Family 5

The proband (I-1) died suddenly, and an autopsy revealed right ventricular hypertrophy and dilatation. Genetic analysis identified a variant of uncertain significance c.10401C>G (p.Phe3467Leu) in the *ANK2* gene, which is associated with cardiac rhythm abnormalities, including LQTS. While the penetrance of pathogenic variants in the *ANK2* gene varies, Joshi et al. indicate its significant role in arrhythmogenic cardiac diseases (Joshi et al., 2024).

A second uncertain variant c.35T>C (p.Ile12Thr), in the *PLN* gene, linked to cardiomyopathies, was also detected in the proband (ClinVar NCBI). Pathogenic variants in the *PLN* gene are known to impair cardiac muscle function, increasing the risk of arrhythmogenic and dilated cardiomyopathy, and ventricular arrhythmias (van der Heide et al., 2024).

The proband’s son (II-2) carries the *ANK2* variant but remains asymptomatic. His other son (II-3) experiences palpitations; however, the variant has not been detected. Daughter (II-1) is healthy and does not carry the variant. The proband’s wife (I-2) served as a healthy control in the study. The *PLN* gene variant identified in the proband was not detected in any of the offspring or the proband’s wife. The risk of sudden cardiac death in offspring depends on the presence of these genetic variants, with *ANK2* carriers at increased risk for arrhythmias.

The proband’s family has an elevated heart disease risk, particularly the son (II-2) with the *ANK2* variant. Ongoing cardiac monitoring, genetic counselling, and preventive measures are crucial to reducing the risk of severe cardiac events, including sudden cardiac death (Ackerman et al., 2011).

### 3.6 Family 6

The proband (II-2) died suddenly at 40, with autopsy findings of hypertrophic cardiomyopathy (HCM), noncompaction cardiomyopathy, and arrhythmias. Genetic testing identified a variant of uncertain significance c.819T>A (p.Cys273Ter) in the *FHL1* gene. *FHL1* encodes a protein essential for sarcomere function and cardiac and skeletal muscle biomechanics, with pathogenic variants linked to neuromuscular and cardiac diseases, including HCM and arrhythmias (Hespe et al., 2025). Penetrance of *FHL1* pathogenic variants is incomplete, influenced by age and other genetic factors (Koutsofti et al., 2024).

The proband’s brother (II-1) has been diagnosed with HCM and palpitations. He carries two variants of uncertain significance: c.819T>A in the *FHL1* gene and c.4978A>G (p.Ile1660Val) in the *SCN5A* gene. *SCN5A* pathogenic variants have been associated with arrhythmogenic disorders, including long QT syndrome (LQTS) and Brugada syndrome, with incomplete penetrance and variable onset (Walsh et al., 2025). These conditions typically manifest during childhood or adolescence and are associated with a high lifelong risk of cardiac events (Marchal et al., 2024).

The proband’s son (III-1) exhibits symptoms consistent with hypertension; however, he does not carry any of the identified variants. His other son (III-2) is healthy and does not possess these variants either. The proband’s wife served as a healthy control in the study.

The risk of sudden cardiac death in the proband’s offspring depends on the presence of pathogenic variants and their level of penetrance. Carriers of *FHL1* and *SCN5A* variants face an increased risk of developing cardiomyopathies and arrhythmias (Hespe et al., 2025; Walsh et al., 2025).

### 3.7 Family 7

The proband (II-1), who has an implantable cardioverter-defibrillator (ICD) due to ventricular tachycardia and arrhythmias, carries a variant of uncertain significance, c.2989G>A (p.Ala997Thr) in the *SCN5A* gene, inherited from his mother (I-2). Additionally, he inherited a *PKP2* variant of uncertain significance, c.2431C>G (p.Arg811Gly), from his father (I-1). While *PKP2* pathogenic variants are commonly associated with autosomal dominant arrhythmogenic right ventricular cardiomyopathy (ARVC), the c.2431C>G variant from his father did not manifest any arrhythmic signs. The penetrance of *PKP2* pathogenic variants varies but is typically higher in families with a history of cardiac events, with onset often in middle age or earlier in cases of severe pathogenic variants (Yang et al., 2024).

Among the proband’s three children, his daughter (III-1) inherited both *SCN5A* and *PKP2* variants, increasing her risk of arrhythmogenic disorders and cardiomyopathies. His sons (III-2, III-3) carry only the *PKP2* variant and remain asymptomatic, suggesting a lower risk of severe cardiac disease.

Regular cardiac follow-up is recommended for all offspring, with a particular focus on the daughter (III-1) due to a higher risk of developing clinical symptoms and sudden cardiac death.

## 4 Discussion

This study proposes an alternative approach to molecular genetic testing in families with cardiopathies and arrhythmic syndromes based on comprehensive clinical and molecular genetic diagnostics as part of routine testing. Panel assays (custom or commercial) have been shown to provide higher coverage and sensitivity than WES and WGS, particularly for well-defined clinically relevant genes. The thorough testing of gene variants, including VUS, in all willing family members, improves segregation analysis in the affected families compared to testing only certain gene variants found in the proband. This process enables faster risk stratification and clinical follow-up of at-risk family members, facilitating improved disease prevention and personalised patient management, and could therefore be beneficial in routine molecular genetic testing.

Segregation analysis, including more detailed testing of gene variants even in asymptomatic family members, can help clarify the genetic aetiology of these diseases, which may be ambiguous within individual families, thereby allowing a more accurate risk assessment for family members. The analysis revealed carriage of pathogenic variants for rare diseases, which has important implications for genetic counselling and family planning. Additionally, pathogenic variants linked to arrhythmic syndromes were identified in some patients originally tested for other cardiac conditions.

However, to reliably determine the true pathogenic potential of a given variant, segregation analysis must be integrated with other approaches, including functional studies and long-term clinical follow-up. The prevalence of variants of uncertain significance was high, which made interpretation of the results difficult. In some cases, though, it may be possible to retrospectively reclassify the result if data from functional studies and segregation analyses are available (Walsh et al., 2024).

In the analysed families, the segregation of genetic variants in genes such as *RBM20* and *SCN5A* is essential to confirm their association with the disease. Segregation analysis thus contributes to a more comprehensive and refined diagnostic outcome, allowing for more precise genetic counselling and improved risk prediction for asymptomatic individuals within these families. This, in turn, supports more effective personalised monitoring and prevention of major cardiac events. In cases involving genes such as *RBM20*, it is crucial to offer regular cardiological evaluation to family members who carry pathogenic or likely pathogenic variants, even if they remain asymptomatic.

In addition, combinations of variants in two genes, *ACTC1* and *CACNA1C*, were identified, and their segregation was observed in association with a modified disease phenotype. This points to a possible digenic or oligogenic heterozygosity in certain cardiogenetic disorders, which could be influenced by the interaction of multiple genetic factors. In a report, either compound, digenic, or oligogenic inheritance pattern was described in 5%–16% of cases (Gómez et al., 2016; McVeigh et al., 2019). Further research may reveal whether specific combinations of variants in multiple genes have an additive or even synergistic effect on disease pathogenesis.

Regarding available guidelines, there have been ESC Guidelines for the management of cardiomyopathies (Arbelo et al., 2023). These refer to sequencing as a possible indication for segregation analysis particularly to inform interpretation of a variant of uncertain significance found in an affected individual, or the affected relatives (Arbelo et al., 2023). Further, a recent consensus by the ESC Council of Cardiovascular Genomics (Elliott et al., 2025) states that testing of a ‘hot’ VUS in a family may be justified when co-segregation of an appropriate phenotype adds the key contributor to the interpretation of pathogenicity. Importantly, the consensus team suggests that for most cardiomyopathies, the clinical risk of overestimating the significance of a VUS is less than that of underestimating a LP/P variant (Elliott et al., 2025).

Furthermore, identified genetic variants can be published in international databases, such as ClinVar (NCBI) and the Human Gene Mutation Database (HGMD). Variants detected in genes such as *DSG2* and *RBM20* from our study will be submitted to ClinVar (NCBI), where they can serve as a valuable resource for other laboratories and contribute to the global effort in variant classification and interpretation. Sharing data on genetic variants allows continuous reassessment of their clinical significance. This process is fundamental for variants of uncertain clinical significance, where additional evidence from other families or functional studies may provide further validation of their pathogenicity or benign nature.

Lastly, close collaboration between cardiologists, clinical geneticists, molecular biologists, and bioinformatic specialists contributes to the accurate diagnosis and evaluation of genetic variants, as well as effective patient management (McVeigh et al., 2019). Unfortunately, the ability to form such a multidisciplinary team is limited by the capacity of a certain workplace. Additionally, extensive testing of asymptomatic family members may bring about anxiety or worry about the risk to children if certain variants are found. Practitioners should be precise and empathetic when discussing the possible risks and implications of variants of uncertain significance. Nevertheless, an accurate diagnosis can facilitate improved disease management and appropriate treatment, whereas a lack thereof may deprive patients of the best care (McAllister et al., 2007).

## 5 Conclusion

In this study, we describe an alternative design for extensive family-based NGS panel testing compared to proband-only NGS panel sequencing. We would like to present this modified diagnostic model for consideration by others, as the use of NGS to screen the whole family may significantly reduce the time to diagnosis and improve risk stratification of all family members. This approach streamlines identification of at-risk carriers, allowing for early individualized preventive measures and targeted treatment. Finally, yet importantly, the greater amount of data generated from testing entire families may be valuable for sharing in databases and may further the understanding of variants’ significance.

## Data Availability

The data used for this study are available on request from the corresponding author. Data are not freely available due to privacy and ethical restrictions. Anonymized data of the subjects included in this study are available upon request.

## References

[B1] Ackerman M. J. Priori S. G. Willems S. Berul C. I. Brugada R. Calkins H. (2011). HRS/EHRA expert consensus statement on the state of genetic testing for the channelopathies and cardiomyopathies: this document was developed as a partnership between the heart rhythm society (HRS) and the european heart rhythm association (EHRA). Ep Eur. 13 (8), 1077–1109. 10.1093/europace/eur245 21810866

[B2] Angelova P. Stoyanov N. Velchev V. Atemin S. Sleptsova M. Todorov Т. (2024). Molecular-genetic profile in patients with cardiomyopathy in Bulgaria. Bulg. Cardiol. 30 (2), 83–105. 10.3897/bgcardio.30.e127156

[B3] Arbelo E. Protonotarios A. Gimeno J. R. Arbustini E. Barriales-Villa R. Basso C. (2023). 2023 ESC guidelines for the management of cardiomyopathies. Eur. Heart J. 44 (37), 3503–3626. 10.1093/eurheartj/ehad194 37622657

[B4] Cipriano L. Piscopo R. Aiello C. Novelli A. Iolascon A. Piscopo C. (2024). Expanding the phenotype of the CACNA1C-Associated neurological disorders in children: systematic literature review and description of a novel mutation. Children 11 (5), 541. 10.3390/children11050541 38790536 PMC11119747

[B5] Das S. Seth S. (2021). Familial dilated cardiomyopathy with RBM20 mutation in an Indian patient: a case report. Egypt. Heart J. 73 (1), 47. 10.1186/s43044-021-00165-6 34021826 PMC8140951

[B6] Elliott P. Schunkert H. Bondue A. Behr E. Carrier L. Van Duijn C. (2025). Integration of genetic testing into diagnostic pathways for cardiomyopathies: a clinical consensus statement by the ESC council on cardiovascular genomics. Eur. Heart J. 46 (4), 344–353. 10.1093/eurheartj/ehae747 39673718

[B7] García‐Padilla C. Lozano-Velasco E. García‐López V. Aránega A. Franco D. García‐Martínez V. (2024). MiR-1 as a key epigenetic regulator in early differentiation of cardiac sinoatrial region. Int. J. Of Mol. Sci. 25 (12), 6608. 10.3390/ijms25126608 38928314 PMC11204236

[B8] Giammarino L. Bains S. Louradour J. Nimani S. Alerni N. Tester D. J. (2024). *In vivo* KCNQ1-suppression-replacement gene therapy in transgenic LQT1 rabbits restores a physiological QT interval at baseline and under catecholamine infusion. Ep Eur. 26 (Suppl. ment_1). 10.1093/europace/euae102.605

[B9] Gómez J. Reguero J. R. Coto E. (2016). The ups and downs of genetic diagnosis of hypertrophic cardiomyopathy. Rev. Española De. Cardiol. 69 (1), 61–68. 10.1016/j.rec.2015.10.001 26654849

[B10] Greve J. N. Schwäbe F. V. Taft M. H. Manstein D. J. (2024). Biochemical characterization of cardiac α‐actin mutations A21V and D26N implicated in hypertrophic cardiomyopathy. Cytoskeleton 81 (12), 815–831. 10.1002/cm.21852 38459932 PMC11615838

[B11] Hespe S. Waddell A. Asatryan B. Owens E. Thaxton C. Adduru M. L. (2025). ClinGen hereditary cardiovascular disease gene curation expert panel: reappraisal of genes associated with hypertrophic cardiomyopathy. Medrxiv Cold Spring Harb. Lab. 85 (7). 10.1101/2024.07.29.24311195 39971408 PMC12079304

[B12] Joshi J. Schwieterman N. Smole N. Guo S. Wan X. Ramirez‐Navarro A. (2024). 473 application of human induced pluripotent stem cell-derived cardiomyocytes (iPSC-CMs) for modeling of Ankyrin-2 p.R990Q variant-induced ventricular arrhythmia and personalized medicine. J. Of Clin. And Transl. Sci. 8 (s1), 139–140. 10.1017/cts.2024.401

[B13] Kathiresan S. Srivastava D. (2012). Genetics of human cardiovascular disease. Cell 148 (6), 1242–1257. 10.1016/j.cell.2012.03.001 22424232 PMC3319439

[B14] Koutsofti C. Ioannides M. Polydorou C. Papagregoriou G. Malatras A. Michael G. (2024). Massive parallel DNA sequencing of patients with inherited cardiomyopathies in Cyprus and suggestion of digenic or oligogenic inheritance. Genes 15 (3), 319. 10.3390/genes15030319 38540378 PMC10970479

[B15] Lacaze P. Sebra R. Riaz M. Ingles J. Tiller J. Thompson B. A. (2021). Genetic variants associated with inherited cardiovascular disorders among 13,131 asymptomatic older adults of European descent. Npj Genomic Med. 6 (1), 51. 10.1038/s41525-021-00211-x 34135346 PMC8209162

[B16] Lukas Laws J. Lancaster M. C. Ben Shoemaker M. Stevenson W. G. Hung R. R. Wells Q. (2022). Arrhythmias as presentation of genetic cardiomyopathy. Circulation Res. 130 (11), 1698–1722. 10.1161/circresaha.122.319835 35617362 PMC9205615

[B17] Marchal G. A. Rivaud M. R. Wolswinkel R. Basso C. van Veen T. A. B. Bezzina C. R. (2024). Genetic background determines the severity of age-dependent cardiac structural abnormalities and arrhythmia susceptibility in Scn5a-1798insD mice. Ep Eur. 26 (6), euae153. 10.1093/europace/euae153 38875491 PMC11203918

[B18] McAllister M. Davies L. Payne K. Nicholls S. Donnai D. MacLeod R. (2007). The emotional effects of genetic diseases: implications for clinical genetics. Am. J. Of Med. Genet. Part A 143A (22), 2651–2661. 10.1002/ajmg.a.32013 17937446

[B19] McVeigh T. P. Kelly L. J. Whitmore E. Clark T. Mullaney B. Barton D. E. (2019). Managing uncertainty in inherited cardiac Pathologies—an international multidisciplinary survey. Eur. J. Of Hum. Genet. 27 (8), 1178–1185. 10.1038/s41431-019-0391-8 30979968 PMC6777443

[B20] Morgat C. Fressart V. Porretta A. P. Neyroud N. Messali A. Temmar Y. (2024). Genetic characterization of KCNQ1 variants improves risk stratification in type 1 long QT syndrome patients. Ep Eur. 26 (6), euae136. 10.1093/europace/euae136 38825991 PMC11203906

[B21] Prawitt D. Eggermann T. (2024). Molecular mechanisms of human overgrowth and use of omics in its diagnostics: chances and challenges. Front. Genet. 15, 1382371. 10.3389/fgene.2024.1382371 38894719 PMC11183334

[B22] Stava T. T. Berge K. E. Haugaa K. H. Smedsrud M. K. Leren T. P. Bogsrud M. P. (2024). Molecular genetics in 1991 arrhythmia probands and 2782 relatives in Norway: results from 17 years of genetic testing in a national laboratory. Clin. Genet. 106 (5), 585–602. 10.1111/cge.14593 39073097

[B23] Tukker M. A. Te Rijdt W. P. Hajdarpašić A. Çalişkan K. (2023). Malignant arrhythmias and advanced heart failure in patients with RBM20 cardiomyopathy: a single center cohort study and review of the literature. Eur. Heart J. 44 (Suppl. ment_2), ehad655. 10.1093/eurheartj/ehad655.1804

[B24] van der Heide M. Y. C. Verstraelen T. E. Mahmoud B. van Drie E. de Brouwer R. Proost V. M. (2024). The impact of comorbidities and substance use on heart failure events and major ventricular arrhythmias in phospholamban p.(Arg14del) positive individuals. Ep Eur. 26 (Suppl. ment_1). 10.1093/europace/euae102.640

[B25] Walsh N. Cooper A. Dockery A. O’Byrne J. J. (2024). Variant reclassification and clinical implications. J. Of Med. Genet. 61 (3), 207–211. 10.1136/jmg-2023-109488 38296635

[B26] Walsh R. Mauleekoonphairoj J. Mengarelli I. Bosada F. M. Verkerk A. O. van Duijvenboden K. (2025). A rare noncoding enhancer variant in *SCN5A* contributes to the high prevalence of Brugada syndrome in Thailand. Circulation 151 (1), 31–44. 10.1161/circulationaha.124.069041 39391988 PMC11670919

[B27] Wilde A. A. M. Semsarian C. Márquez M. F. Sepehri Shamloo A. Ackerman M. J. Ashley E. A. (2022). European heart rhythm association (EHRA)/heart rhythm society (HRS)/asia Pacific heart rhythm society (APHRS)/latin American heart rhythm society (LAHRS) expert consensus statement on the state of genetic testing for cardiac diseases. J. Of Arrhythmia 38 (4), 491–553. 10.1002/joa3.12717 35936045 PMC9347209

[B28] Yan J. Zhong X. Zhao Y. Wang X. (2024). Role and mechanism of miRNA in cardiac microvascular endothelial cells in cardiovascular diseases. Front. Cardiovasc. Med. 11, 1356152. 10.3389/fcvm.2024.1356152 38545341 PMC10966125

[B29] Yang Z. H. J. Wu I. Zeng A. Greer-Short A. Aycinena J. A. Tefera A. E. (2024). PKP2 gene therapy reduces ventricular arrhythmias, reverses ventricular remodeling, improves heart function, and reduces mortality in a mouse model of arrhythmogenic right ventricular cardiomyopathy. Ep Eur. 26 (Suppl. ment_1), euae102.606. 10.1093/europace/euae102.606

[B30] Ylipää J. Andersson T. (2024). Genetic analysis and family screening for dilated cardiomyopathy: a retrospective analysis of the stepwise pedigree approach. Scand. Cardiovasc. J. 58 (1), 2379356. 10.1080/14017431.2024.2379356 39046218

