# Supplementary material for: Family-based NGS panel testing of cardiopathies and arrhythmic syndromes
Source: Front Genet. 2025 Nov 3;16:1677311. doi: 10.3389/fgene.2025.1677311 (PMC12640736; doi:10.3389/fgene.2025.1677311)
Supplement: Supplementary file 1 [file Table2.docx]

**Table S2**: List of Genes Analyzed by NGS

| *A2ML1, ABCC9, ABL1, ACADVL, ACAN, ACD, ACE, ACTA2, ACTC1, ACTN2, ACVR1, ACVRL1, ADAMTS10, ADAMTS17, ADAMTS2, ADAMTSL4, AEBP1, AFF4, AGGF1, AGL, AKAP9, AKT1, AKT2, AKT3, ALDH18A1, ALG10B, ALMS1, ALPK3, ALX1, ALX3, ALX4, ANAPC1, ANGPTL6, ANK1, ANK2, ANKRD1, ANKRD11, ANOS1, ANTXR2, APC2, APOA1, APOA2, APOC2, APOC3, AR, ARHGAP31, ARHGEF17, ARID1A, ARID1B, ARID2, ARNT2, ARX, ASCC1, ASCL1, ASPH, ASXL1, ASXL3, ATP6V0A2, ATP6V1A, ATP6V1E1, ATP7A, ATP7B, ATR, ATRIP, B2M, B3GALT6, B3GAT3, B4GALT7, BAG3, BGN, BICD2, BMP1, BMP2, BMPR1B, BMPR2, BRAF, BRCC3, BRD4, BRWD3, BTD, BTRC, C1R, C1S, CACNA1C, CACNA1D, CACNA2D1, CACNB2, CALM1, CALM2, CALM3, CALR3, CASK, CASQ2, CASR, CAV3, CAVIN4 (MURC), CBL, CBS, CCDC141, CCDC8, CCER2, CCM2, CCND2, CD96, CDC45, CDC6, CDH2, CDH3, CDK4, CDKL5, CDKN1C, CDKN2A, CDKN2B, CDT1, CENPE, CENPJ, CEP120, CEP152, CEP63, CFTR, CHD4, CHD7, CHRM2, CHST14, CITED2, CNOT3, CNTNAP2, COG4, COL11A1, COL11A2, COL12A1, COL17A1, COL1A1, COL1A2, COL22A1, COL2A1, COL3A1, COL4A1, COL4A2, COL5A1, COL5A2, COL7A1, COL9A1, COL9A2, COL9A3, COLEC10, COLEC11, COMP, CREB3L1, CREBBP, CRELD1, CRTAP, CRYAB, CSRP3, CST3, CTC1, CTF1, CTNNA3, CTRC, CUL4B, CUL7, CYP11B1, CYP17A1, DCX, DEAF1, DES, DHCR24, DHCR7, DIS3L2, DKC1, DLG4, DLL4, DLX5, DLX6, DMD, DNA2, DNAJC19, DNM2, DNMT3A, DOCK6, DOK7, DOLK, DPF2, DPM3, DSC2, DSE, DSG2, DSP, DTNA, DUSP6, DYNC2H1, DYNC2LI1, ECE1, ECEL1, EDN3, EDNRA, EDNRB, EED, EFEMP2 (FBLN4), EFNB1, ELN, EMD, EMILIN1, ENG, EOGT, EP300, EPB42, EPG5, EPS15L1, ERF, ESCO2, EVC, EVC2, EXT1, EXT2, EYA4, EZH2, FBLN1, FBLN5, FBN1, FBN2, FEZF1, FGA, FGD1, FGF12, FGF16, FGF17, FGF8, FGFR1, FGFR2, FGFR3, FHL1, FHL2, FHOD3, FKBP10, FKBP14, FKTN, FLII, FLNA, FLNB, FLNC, FLRT3, FOXE3, FOXF1, FOXG1, FREM1, FSHB, GAA, GABBR2, GATA1, GATA4, GATA5, GATA6, GATAD1, GDF2, GDF3, GDF5, GDF6, GDNF, GJA1, GJA5, GLA, GLI1, GLI2, GLI3, GMNN, GNAS, GNRH1, GNRHR, GORAB, GPC3, GPC4, GPD1L, GRHL3, GSN, GUCY1A3/GUCY1A1, HAND1, HCN4, HDAC8, HESX1, HOXD13, HPGD, HRAS, HS6ST1, HSD3B2, IDH1, IDH2, IFITM5, IFT122, IFT140, IFT172, IFT43, IFT52, IFT80, IGF1, IGF2, IHH, IKBKG, IL17RD, ILK, IQCE, IQSEC2, IRF6, ITGA6, ITGB4, JAG1, JPH2, JUP, KAT6B, KCNA5, KCND2, KCND3, KCNE1, KCNE2, KCNE3, KCNE5 (KCNE1L), KCNH2, KCNJ2, KCNJ5, KCNJ8, KCNQ1, KCTD1, KDELR2, KDM6A, KIAA0586, KIF22, KIF2A, KIF5C, KISS1, KISS1R, KIT, KITLG, KLF10, KLHL7, KMT2A, KMT2D, KRAS, KRIT1, KRT14, KRT5, L1CAM, LAMA3, LAMA4, LAMB3, LAMC2, LAMP2, LDB3, LEP, LEPR, LEPRE1 (P3H1), LEPROT, LHB, LHX3, LHX4, LIG4, LMBR1, LMNA, LMX1B, LOX, LOXL2, LRP1, LRP5, LRRC10, LTBP2, LTBP4, LYZ, LZTR1, MAGEL2, MAP2K1, MAP2K2, MASP1, MAT2A, MAU2, MBD5, MBTPS2, MC2R, MC3R, MC4R, MCM5, MCPH1, MECP2, MED12, MEFV, MEGF8, MEIS2, MEOX1, MESD, MFAP5, MIB1, MIB2, MID1, MRAS, MSX2, MTOR, MUSK, MVK, MYBPC1, MYBPC3, MYH11, MYH3, MYH6, MYH7, MYH7B, MYH8, MYL2, MYL3, MYL4, MYLK, MYLK2, MYO18B, MYOD1, MYOM1, MYOT, MYOZ2, MYPN, MYRF, NALCN, NBAS, NBN, NCOA6, NEBL, NEK1, NEK9, NEXN, NF1, NF2, NFIX, NHP2, NIN, NIPBL, NKX2-5, NKX2-6, NOD2, NOG, NOP10, NOS1AP, NOTCH1, NOTCH2, NPPA, NR0B1, NR2F2, NRAP, NRAS, NRTN, NRXN1, NSD1, NSMCE2, NSMF, NSUN2, NTNG1, NTRK1, NUP155, NUP88, OBSCN, OBSL1, OFD1, ORC1, ORC4, ORC6, OTX2, P4HA1, PAFAH1B1, PAH, PARN, PAX3, PCNT, PDCD10, PDE3A, PDGFRB, PDLIM3, PHACTR1, PHF6, PHOX2B, PIEZO2, PIGA, PIGN, PIK3CA, PIK3R1, PIK3R2, PKD1, PKD2, PKP2, PLK4, PLN, PLOD1, PLOD2, PLS3, POLR1C, POLR1D, POMC, POR, POU1F1, PPA2, PPCS, PPIB, PPP1CB, PRDM16, PRDM5, PRF1, PRKAG2, PRKAR1A, PRKG1, PRMT7, PROK2, PROKR2, PROP1, PTCH1, PTCH2, PTEN, PTH1R, PTHLH, PTPN11, PYCR1, RAB23, RAD21, RAF1, RAI1, RANGRF, RAP1A, RAP1B, RAPSN, RASA1, RASA2, RASA3, RBBP8, RBM20, RBM8A, RBPJ, RECQL4, RELN, RET, RIN2, RIT1, RLIM, RNF135, RNF213, ROBO4, ROR2, RPL11, RPL15, RPL26, RPL35A, RPL5, RPS10, RPS17, RPS19, RPS24, RPS26, RPS28, RPS29, RPS6KA3, RPS7, RRAS, RRAS2, RTEL1, RYR2, SALL4, SAMD9, SBDS, SC5D, SCN10A, SCN1B, SCN2B, SCN3B, SCN4B, SCN5A, SCN9A, SCO2, SDHA, SEC24D, SEM1, SEMA3A, SEMA3C, SEMA3D, SEMA3E, SERPINF1, SERPINH1, SETD2, SETD5, SGCD, SGCG, SH2B1, SHH, SHOC2, SHOX, SIM1, SKI, SLC18A3, SLC22A5, SLC26A2, SLC2A10, SLC2A2, SLC39A13, SLMAP, SMAD2, SMAD3, SMAD4, SMAD6, SMARCA2, SMARCA4, SMARCB1, SMARCC2, SMARCE1, SMC1A, SMC3, SNAI2, SNTA1, SOS1, SOS2, SOST, SOX10, SOX11, SOX2, SOX3, SOX4, SOX9, SP7, SPARC, SPECC1L, SPINK1, SPRED1, SPRY4, SPTA1, SPTB, SRCAP, STAG2, STAR, STXBP1, SUFU, SYNE2, TAB2, TAC3, TACR3, TAF1A, TAZ, TBCK, TBX1, TBX20, TBX4, TBX5, TCAP, TCF12, TCF4, TCOF1, TCTEX1D2/DYNLT2B, TCTN3, TECRL, TENT5A (FAM46A), TERC, TERT, TFAP2A, TGFB2, TGFB3, TGFBR1, TGFBR2, TGFBR3, THSD1, TINF2, TLL1, TMEM38B, TMEM43, TMPO, TNFRSF13B, TNNC1, TNNI2, TNNI3, TNNI3K, TNNT2, TNNT3, TNXB, TP63, TPM1, TPM2, TRAIP, TRDN, TRIM54, TRIM55, TRIM63, TRIP11, TRPM4, TRPV4, TSR2, TTC21B, TTN, TTR, TUBA8, TUBB, TUBB2A, TUBB2B, TUBB3, TUBG1, TWIST1, TXNRD2, UBE3A, UGT1A1, UNC80, UPF3B, VCAN, VCL, VPS13B, WDR11, WDR19, WDR34/DYNC2I2, WDR35, WDR60/DYNC2I1, WNT1, WNT10B, WNT7A, WRAP53, YWHAE, YY1AP1, ZDHHC9, ZEB2, ZFPM2, ZIC1, ZNF141, ZNF469* |
| --- |
